# RmsdXNA: RMSD prediction of nucleic acid-ligand docking poses using machine-learning method

**DOI:** 10.1093/bib/bbae166

**Published:** 2024-05-01

**Authors:** Lai Heng Tan, Chee Keong Kwoh, Yuguang Mu

**Affiliations:** Interdisciplinary Graduate School, Nanyang Technological University, 61 Nanyang Drive, 637335 Singapore, Singapore; School of Computer Science and Engineering, Nanyang Technological University, 50 Nanyang Avenue, 639798 Singapore, Singapore; School of Biological Sciences, Nanyang Technological University, 60 Nanyang Drive, 637551 Singapore, Singapore

**Keywords:** nucleic acid, machine learning, RMSD prediction, molecular docking

## Abstract

Small molecule drugs can be used to target nucleic acids (NA) to regulate biological processes. Computational modeling methods, such as molecular docking or scoring functions, are commonly employed to facilitate drug design. However, the accuracy of the scoring function in predicting the closest-to-native docking pose is often suboptimal. To overcome this problem, a machine learning model, RmsdXNA, was developed to predict the root-mean-square-deviation (RMSD) of ligand docking poses in NA complexes. The versatility of RmsdXNA has been demonstrated by its successful application to various complexes involving different types of NA receptors and ligands, including metal complexes and short peptides. The predicted RMSD by RmsdXNA was strongly correlated with the actual RMSD of the docked poses. RmsdXNA also outperformed the rDock scoring function in ranking and identifying closest-to-native docking poses across different structural groups and on the testing dataset. Using experimental validated results conducted on polyadenylated nuclear element for nuclear expression triplex, RmsdXNA demonstrated better screening power for the RNA-small molecule complex compared to rDock. Molecular dynamics simulations were subsequently employed to validate the binding of top-scoring ligand candidates selected by RmsdXNA and rDock on MALAT1. The results showed that RmsdXNA has a higher success rate in identifying promising ligands that can bind well to the receptor. The development of an accurate docking score for a NA–ligand complex can aid in drug discovery and development advancements. The code to use RmsdXNA is available at the GitHub repository https://github.com/laiheng001/RmsdXNA.

## INTRODUCTION

Therapeutic treatments have typically focused on modifying protein targets [1, 2], but there is a growing acknowledgment of the critical role of ribonucleic acid (RNA) and deoxyribonucleic acid (DNA) molecules in numerous biological processes and disease pathways [3–6]. Dysfunctions in RNA and DNA that can cause diseases [7–9], such as cancer, neurological problems and viral infections [10, 11], can be treated using small chemicals. As our understanding of NA increases, especially with developments in computational methodologies [12, 13] and structure-solving technologies [14], targeting NA for drug development shows promise in the field of molecular medicine.

Currently, however, solving the structure of NA complexes using experimental methods remains a challenge. As an alternative, computational methods, including molecular docking, can enhance the understanding of NA complexes and expedite the development of robust drug discovery models [15–17]. Recent development of such docking tools for NA–ligand complexes includes rDock [18], NLDock [19] and RLDOCK [20]. Molecular docking involves predicting the binding orientation and affinity between a small molecule (ligand) and a target protein or NA (receptor). Its precision depends on the reliability of the scoring functions [21, 22], which is crucial for saving time and cost for drug discovery [23, 24]. However, accurate modelling of NA structures remains challenging because of its complexity and flexibility [25]. While the MM-PBSA [26] and free-energy perturbation methods [27] can accurately select stronger ligand binders compared to docking scoring functions, they are time-consuming and not suitable for high-throughput docking or virtual screening (VS) applications.

Integrating machine learning (ML) into docking algorithms can improve the accuracy and efficiency of computational tools [28–30]. Numerous ML algorithms that outperform a broad range of classical scoring functions have been successfully utilized in protein systems [31]. However, these scoring functions often cannot be applied directly to NA complexes [32, 33]. The limited availability of high-quality training datasets for NA complex structures poses a significant obstacle to the generation of accurate and robust models [34]. Hence, the exploration of alternative feature extraction and ML algorithms methods is essential for addressing these challenges.

Although not as common as protein–ligand systems, two recent ML-based scoring functions have been developed to improve scoring functions for RNA–ligand interactions. RNAPosers [35] is trained on 80 RNA–ligand complexes to classify if the ligand pose is native-like. On the other hand, AnnapuRNA [36] is trained on 131 RNA–ligand complexes to develop a scoring function for predicting binding poses. Both methods demonstrated improved performance compared to existing tools for pose identification. However, the small dataset used for training the models can lead to overfitting and hinder the model’s performance for unseen docking scenarios. In addition, both methods use interaction features involving unmodified RNA residues only, neglecting crucial interactions involving modified atoms, which can alter the chemical properties of NA [37]. A diversified model should also be applicable to some important NA–ligand complexes, such as metal complex [38] or short peptide ligands [39], and RNA–DNA hybrid receptors [40]. Developing a ML-based scoring function suitable for NAs (both DNA and RNA) and structures with modified residues seems feasible due to their structural similarity.

In this work, a new ML-based regression model, RmsdXNA, was developed for accurately predicting the RMSD of the ligand poses for NA–ligand complex, which can also serve as the docking score. Using a larger dataset of 980 PDB structures than previous studies aims to improve generalizability. As the number of NA structures is still relatively small compared to proteins, using similar distance-based fingerprints from Wang et al. [41] with small feature dimension is feasible for RmsdXNA. Further modifications to the atom representation and the machine learning algorithm were performed to better suit NA–ligand datasets. RmsdXNA is applicable to diverse complexes involving different types of NA receptors (RNA, DNA, RNA–DNA hybrid, and NA with modified residues) and ligands, including metal complexes and short peptides. The best performing RmsdXNA model achieved an average Pearson correlation coefficient (PCC) of 0.64531 $\pm $ 0.36262 and Spearman’s rank correlation coefficient (SRCC) of 0.58465 $\pm $ 0.35195 for each ligand. RmsdXNA outperforms the rDock [18] scoring function in its ability to rank and identify closest-to-native poses across different structural groups and on the testing dataset. This was also validated using the in-vitro experimental results by Swain et al. [42] on PAN ENE triple helix RNA. For MALAT1 virtual screening, MD simulations revealed a higher success rate for RmsdXNA in identifying the most promising ligands. These findings solidify RmsdXNA as a robust and effective scoring tool for pose identification and ligand screening in drug discovery applications.

## MATERIALS AND METHODS

### Dataset preparation

The dataset used in the experiments consists of experimentally solved NA structures obtained from the Nucleic Acid Database (NDB) [43, 44], which contains 11 821 NA structures including both DNA and RNA. PyMol [45] was used to process the structural information. Additional data cleaning and refinement procedures are described in Appendix B. In total, 1434 ligands from 980 NA complexes, with some structures having multiple ligands, were used in the experiment. The use of a large and diverse database allows the model to learn a comprehensive representation of the underlying patterns and relationships in the data, leading to better generalization to unseen examples. The list of PDB structures used is found in Appendix A.

### Docking tools

Each ligand forms a receptor–ligand pair in each complex. Rigid NA-flexible ligand docking was performed using rDocK [18] at the target search box, with the native ligand as the reference ligand. rDock was chosen because it outperforms other docking tools in pose identification for NA complexes [19, 46]. The docking pose search algorithm of rDock is similar to that of AutoDock [47], which uses the Monte Carlo simulated annealing method to generate random poses with unique configurations in each dock attempt. This data augmentation method can mitigate the limitation of the small NA–ligand dataset by increasing the number of datapoints with generated poses. Using rDock, the *rbcavity* command was used to generate the docking volume, with the docking radius set to 10 Å. For each redocking run, 100 runs-per-ligand rDock jobs were performed using the *rbdock* command. Only the docking poses with symmetry-corrected RMSD values $\le $ 10 Å, measured using sPyRMSD [48], were considered as a successful dock and were included in the training dataset. 100 poses were selected from each ligand that had at least 25% successful docking rate (able to generate at least 100 successful dock poses out of 400 dock attempts) to be included into the dataset.

### Feature generation

**Figure 1:**
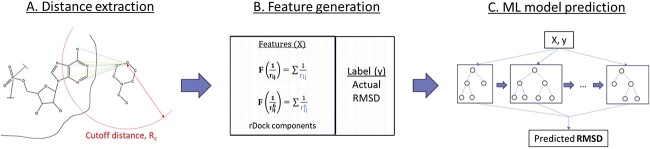
Overall architecture of the RmsdXNA framework. (**A**) Inter-molecular distance between a NA receptor atom and the ligand atom within a cutoff distance, $R_{c}$ is extracted for each generated pose. (**B**) The distance is used to form the $F(\frac{1}{r})$ and $F(\frac{1}{r^{6}})$ terms, which will be concatenate with the rDock scoring components to form the features, *X*. The label, *y* is the actual RMSD of the docked poses. (**C**) XGBoostRegressor is used to train on the dataset predict the RMSD of the docked poses.

The workflow of dataset generation and model training for RmsdXNA to predict the RMSD of the docked poses is shown in Figure 1. For each distinctive interaction between the receptor and docked ligand atoms, the interatomic distance and atom types are used as features. In the context of ML scoring functions for NAs, the quantity of structures within the dataset is notably lower than that of the proteins. Reducing the number of employed features is crucial to prevent overfitting, the curse of dimensionality and data sparsity. Previous studies such as OnionNet [49] and OnionNet2 [50] adopted the count of contact points at each shell as their feature metric, whereas RNAposers [35] employed the receptor’s atom name to generate distance-based features. However, these methodologies create an abundance of features, resulting in an unfavorable high feature-to-instance ratio dataset. On the other hand, the coarse-grain representation of the RNA and ligand structures [51, 52] in AnnapuRNA [36] might sacrifice atomic-level details, especially for non-canonical residues, potentially compromising the model’s ability to capture specific interactions.

In RmsdXNA, the receptor and ligand SYBYL atom type representation is used as the interaction label. This generates fewer features in the dataset and allows the retention of important atomic-level interaction information such as pi-stacking and hydrogen bonding interactions. The remaining challenge involves combining each pairwise distance into a structured dataset suitable for training the ML model, while simultaneously retaining interaction proximity and frequency information. The distance is used to model the intermolecular potential energy between the receptor and the ligand molecule, which is represented by the sum of the coulomb and Lennard-Jones interaction terms, as shown in Equation 1. Such physics-inspired distance features were explored by Wang et al. [41] in protein–ligand interactions. 


(1) \begin{align*} \sum_{i,j=1}^{N}\frac{q_{i}q_{j}}{r_{ij}} +& \varepsilon_{ij}\left[\left(\frac{\sigma_{i}}{r_{ij}}\right)^{12} - \left(\frac{\sigma_{i}}{r_{ij}}\right)^{6}\right] \end{align*}

(2) \begin{align*} & \sum_{i,j=1}^{N}\frac{a}{r_{ij}} + \frac{b}{r_{ij}^{12}} + \frac{c}{r_{ij}^{6}} \end{align*}

The values of the interaction constants are unknown and are factorized to form constants *a*, *b* and *c*. Equation 1 can be simplified to form Equation 2 by factorizing the constants. For a given receptor–ligand structure, the intermolecular distance between 2 atoms, $r_{ij}$, can be easily obtained from the structural coordinates. ML is then used to determine the arbitrary constants. Assuming that there is a correlation between the RMSD and intermolecular forces, the RMSD can be predicted using the above equations. The intuition behind this assumption is that the closer-to-native ligand pose (smaller RMSD) should have stronger intermolecular interaction with NA than other poses. For each docked ligand pose, the interaction feature values between receptor atom $i$ and ligand atom $j$ similar to Equation 2 are represented by $F(f(r_{ij}))$, such that: 


\begin{align*} & F(f(r_{ij}))= \begin{cases} \sum{f(r_{ij})}, & \text{if } r_{ij} \in [0, R_{c}) \\ 0, & \text{otherwise} \end{cases} \end{align*}

where:

$f(r_{ij})$ = fingerprint function, for $f(r_{ij}) \in $ {$\frac{1}{r_{ij}}$, $\frac{1}{r^{6}_{ij}}$,

$\frac{1}{r^{12}_{ij}}$ }$r_{ij}$ = distance between the receptor atom $i$ and ligand atom $j$

$i$ = receptor residue-atom type, represented by $RES_{rec}$

$RES$ = receptor residue type, for $RES \in $ {A, C, G, U/T, N, OTH, Metal}

$rec$ = receptor SYBYL atom type 

$j $ = ligand SYBYL atom type 

$R_c$ =cutoff distance (Å), for $R_{c} \in $ {7, 8, 9, 10}

The NA residues can be classified into five generalized types, namely adenine (A), uracil (U) or thymine (T), cytosine (C), guanine (G), and ribose and phosphate backbone (N). The nucleobases of DNA and modified residue nucleobases are grouped into 4 main RNA nucleobases (A, U, G, C), depending on which nucleobases the residues are mutated from or have the closest structural similarity with (Appendix D), with thymine being in the same group as uracil. The NA SYBYL atom types are determined based on their position on these residues, and atoms in the backbone have residue type ’N’ (Appendix E). The atoms in the modified region of the residues have residue type ’OTH’. These NA atoms and some ligand atoms with low occurrence are grouped by their chemical similarity, as described in Appendix C. The grouping of the residues and atom types reduces the sparsity of the dataset and the number of features in the dataset by binning the columns with a high number of 0-values, hence improving the generalizability of the model and making it applicable to datasets with modified residues. In total, 24 ligand atom types and 26 NA atom types (unique combinations of the residues and its corresponding atom types) were used, resulting in 624 pairs of interactions. Subsequently, columns with all 0 values were removed from the dataset, thus a final 529 pairs of interactions were included.

In the ablation studies performed in Section 3.2, the dataset that yielded the best result used a cutoff distance, $R_{c}$, of 8 Å, along with the combination of the 1088 $F(\frac{1}{r})$ and $F(\frac{1}{r^{6}})$ terms with the 30 rDock scoring function component terms for the features. It is noted that the model can be used to predict the RMSD of poses generated by other docking tools as the rDock scoring function components can also be obtained from the initial poses.

### Machine learning models

A supervised learning algorithm is used to train the ML model to predict the RMSD of docked poses, which can also be utilized as the docking score. For this regression prediction task, the XGBoost algorithm was chosen because of its fast training speed and strong predictive capabilities in comparison with other ML models such as Support Vector Regression, Multi-layer Perceptron, and Random Forest algorithms as shown in Table A4. The superior performance of XGBoost over other algorithms may be due to the sparsity of the dataset. To ensure that datasets with the same PDB ID are not present in both the training and validation datasets simultaneously, systematic sampling is employed on the sorted PDB IDs to select the validating PDB ID at regular intervals. The data points are then split into training and validation datasets by their PDB ID. For each dataset, 100 models with randomized hyperparameter values are trained using five-fold cross-validation to assess the models’ performance across different data subsets, which provides a reliable assessment of the model’s generalization capabilities. $R^{2}$ values (Equation 3) are calculated for each fold. The model with the highest average validating $R^{2}$ across all folds, $R^{2}_{val}$, which indicates a strong correlation between predicted and actual RMSD, was selected as the best model. The model is trained with the learning objective of minimizing the regression squared loss and the hyperparameters of the best model are shown in Appendix F. 


(3) \begin{align*}& R^{2} = 1 - \frac{\sum{(y - \hat{y})^{2}}}{\sum{(y - \bar{y})^{2}}}\end{align*}

where: $y$ = Actual RMSD of pose

$\hat{y}$ = Predicted RMSD of pose

$\bar{y}$ = Average actual RMSD of all poses

### Validation using experimental result and MD simulations

Unlike protein–ligand complexes, there is no CASF-2016 [53] equivalent dataset for evaluating NA complex docking tools. Instead, the comparison of the scoring power of RmsdXNA and rDock is validated on experimental results and using MD simulation.

**Figure A2:**
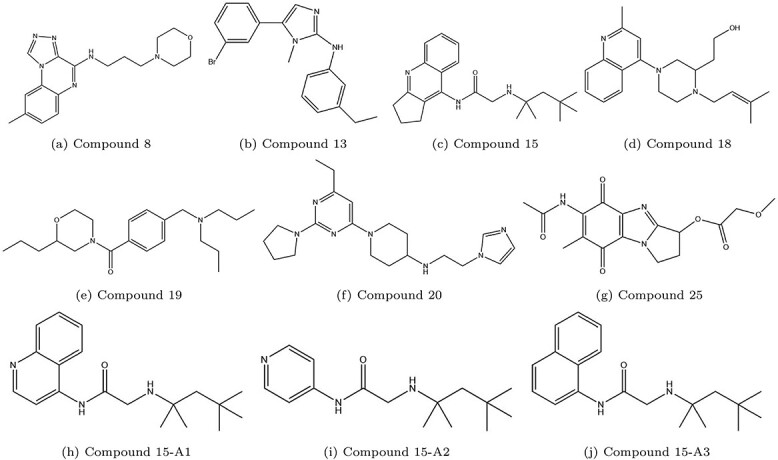
The atom names of residues A, G, C, U/T.

In the microscale thermophoresis (MST) experiment by Swain et al. [42] on $^{GC}$PAN triplex, Compound 15 exhibited the strongest interaction among seven hit compounds (Compounds 8, 13, 15, 18, 19, 20 and 25) identified by small-molecule microarray (SMM) screening. Weak interactions were observed for the 3 Compound 15 modifications (Compounds 15-A1, 15-A2, 15-A3) compared to Compound 15, with Compounds 15-A2 and 15-A3 failing to bind to the RNA. The structures of the compounds are shown in Figure A2. Using the MST results, an ideal docking score that can distinguish strong and weak binders should be able to accurately rank the compounds by their binding affinity and give the best ranking to Compound 15. Rigid receptor-flexible ligand docking of the compounds was performed using rDock on the dinucleotide bulge position of $^{GC}$PAN triplex (PDB ID: 6X5N), generating 100 poses for each compound. Then, RmsdXNA and rDock scores were used to rank the compounds.

MD simulations can offer insights into the dynamic behavior of receptor–ligand complexes, thus providing a more accurate assessment of ligand trajectories stability compared to docking alone [54, 55]. In this VS example, rDock is utilized to generate 20 poses within a 20 Å target search box on the 3’ end of MALAT1 [56, 57] (PDB ID: 4PLX) against two screening compound libraries, Hit2Lead (provided by Chembridge Corp. in San Diego, CA, USA, http://www.hit2lead.com) and Life Chemicals RNA screening library (https://www.lifechemicals.com). From each library, the top 20 ligands with the best rDock and RmsdXNA scores were chosen as the top-scored ligands for each method. Then, MD simulation (described in Appendix H) was conducted on the MALAT1-top ligand complexes to assess the reliability of the docking scoring methods through visual inspection the ligands’ stability in the receptor during the 100 ns trajectory [58].

## RESULTS AND DISCUSSION

### Model performance

**Figure 2:**
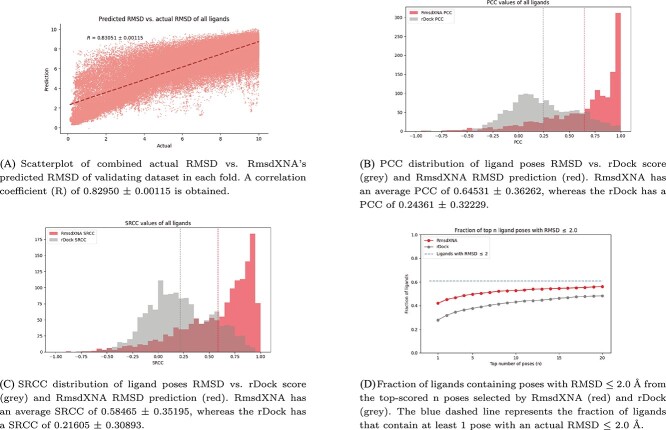
Performance of RmsdXNA and its comparison with rDock on the full validation dataset.

The best model obtained from the hyperparameter tuning procedure had a $R^{2}_{val}$ of 0.68301 $\pm $ 0.02330. The predicted RMSD values of each docked ligand pose in the validation dataset were combined across all folds to obtain the overall predicted RMSD for the entire dataset. Since the validation data remain unseen by the model in each fold, the obtained predictions accurately represent the model’s generalizability. Figure 2(a) shows the comparison of the predicted RMSD with the actual RMSD of the docked poses, which has a strong correlation coefficient (R) of 0.83051 $\pm $ 0.00115. This finding underscores the RmsdXNA’s ability to accurately predict RMSD values.

An effective score function should assign lower scores (better ranking) to the closest-to-native pose, which has a lower actual RMSD than the decoy poses. Therefore, the PCC (Equation 4) and SRCC (Equation 5) between the actual RMSD and scoring functions for each NA–ligand pair, which measure the correlations and ranking capabilities, respectively, are key metrics for evaluating their performance. Figure 2(b) and 2(c) illustrates each ligand’s distribution of the PCC and SRCC values respectively for both scoring methods. RmsdXNA has a higher average PCC and SRCC than rDock. In addition, RmsdXNA outperforms rDock for 1271/1434 (88.6%) and 1254/1434 (87.4%) ligands in terms of PCC and SRCC respectively. This comparison revealed that RmsdXNA has a stronger correlation and ranking capabilities than rDock for most of the ligand structures. 


(4) \begin{align*}& PCC = \frac{\sum{(x-\bar{x})(s-\bar{s})}}{\sqrt{\sum{(x-\bar{x})^{2}}\sum{(s-\bar{s})^{2}}}}\end{align*}

where:

$x$ = Actual RMSD of pose

$\bar{x}$ = Average actual RMSD of all poses

$s$ = Score of pose

$\bar{s}$ = Average score of all poses 


(5) \begin{align*}& SRCC = 1 - \frac{6\sum{(R(x)-R(s))^{2}}}{n(n^{2}-1)}\end{align*}

where:

$R(x)$ = ranking of pose based on RMSD (x)

$R(s)$ = ranking of pose based on score (s)

$n$ = number of poses

The ability of RmsdXNA and rDock to select close-to-native poses, defined by poses with RMSD $\leq $ 2.0, was also analyzed. Figure 2(d) shows that the fraction of ligands that contained close-to-native poses in the top-*n*-scored poses was greater for RmsdXNA. Additionally, out of all the ligands that are able to generate at least 1 close-to-native pose, 69.1%, 81.9% and 86.9% of the ligands for RmsdXNA and 45.9%, 62.0% and 70.8% of the ligands for rDock contained close-to-native poses in the top 1, 5, and 10 selected poses respectively. These results showed that RmsdXNA outperforms the rDock scoring function in pose identification.

### Ablation studies

Ablation experiments were conducted to investigate the impact of various modifications to dataset generation on the performance of the model. The modified dataset was used to train the model using the steps described in Section 2.4. The model with the highest $R^{2}_{val}$ for each modified dataset was subsequently selected and compared across the different modifications.

First, the effect of $R_{c}$ on dataset generation was examined and the results are summarized in Table A5. As $R_{c}$ varies from 4 to 10 Å, more interaction features are extracted and added to the dataset. New features with non-0 values may also be introduced into the dataset, increasing the number of columns of the dataset slightly. Previous studies by Szulc et al. [59] employed cutoff distances of 3.9 and 4.0 Å to detect hydrogen bonding and lipophilic interactions respectively in RNA complexes. However, $R_{c}$ = 8 Å gave the highest $R^{2}_{val}$ of 0.68301 $\pm $ 0.02330. The improvement in the results from $R_{c}$ = 4 to 8 Å may be due to the increase in the number of non-0 values in the dataset and the addition of new information for the model. Beyond certain distances, such as $R_{c}$ = 9 and 10 Å, the added long-range and weak interaction may introduce noise to the data and additional information has insignificant contributions to the specific interaction between NA and ligand. Therefore, finding the optimal cutoff distance is vital for striking the right balance between capturing relevant interactions and avoiding unnecessary complexity in the dataset.

Next, the effect of adding the rDock scoring components, $F(\frac{1}{r})$, $F(\frac{1}{r^{6}})$ and $F(\frac{1}{r^{12}})$ features into the dataset was investigated. By generating datasets that combine these specific features, their contribution to the overall model performance can be evaluated and the best performing combinations can be determined. The results of the model with different combinations of features are shown in Table A6. The best result of $R^{2}_{val}$ = 0.68301 $\pm $ 0.02330 was achieved when the rDock scoring function components were added to the $F(\frac{1}{r})$ and $F(\frac{1}{r^{6}})$ features. The improved prediction accuracy suggests that the rDock components and the generated features complement each other effectively. Additionally, the analysis revealed that the $F(\frac{1}{r^{12}})$ component is less important than the $F(\frac{1}{r})$ and $F(\frac{1}{r^{6}})$ components, as the $R^{2}_{val}$ of the model decreased to 0.67910 $\pm $ 0.01763 when the $F(\frac{1}{r^{12}})$ component was added to the dataset. Wang et al. [41] explained that the short-range repulsive term, $F(\frac{1}{r^{12}})$, is not relevant because of the low occurrence of small-distance interactions in the NA–ligand complex. Although similar distance data are used to generate the feature values of $F(\frac{1}{r})$, $F(\frac{1}{r^{6}})$ and $F(\frac{1}{r^{12}})$, the difference in the results suggests that proper data augmentation of the feature is needed to achieve high accuracy of the model.

Finally, the impact of different atom representation methods when generating the dataset on $R^{2}_{val}$ is summarized in Table A7. The use of the SYBYL atom type to represent receptor and ligand atoms, element type to represent receptor atoms with the ’OTH’ residue type, and other grouping methods mentioned in Appendix C, achieved the highest $R^{2}_{val}$ of 0.68301 $\pm $ 0.02330. This outperformed models that used a full element or SYBYL atom representation with $R^{2}_{val}$ values of 0.66726 $\pm $ 0.02244 and 0.67945 $\pm $ 0.01654 respectively. Compared to DeepRMSD, which uses element atom representation, using the SYBYL atom type can distinguish aromatic and non-aromatic atoms. This allows the model to capture pi-stacking interactions between the NA receptor and ligand which are abundant in NA complexes [60]. Additionally, to handle sparse features involving NA modified residues atoms, binning them with other meaningful features can offer more instances for the model to learn from, while reducing the introduction of biased information.

### Feature importance analysis

Feature importance analysis was conducted to determine the relative importance of individual features in predicting RMSD in the model. The following training process uses the hyperparameter obtained from the best model. For each fold, the model is used to predict the RMSD of the ligands from the modified validating dataset where a specific *ij* pair feature ($F(\frac{1}{r_{ij}})$ and $F(\frac{1}{r^{6}_{ij}})$), or the rDock score component is artificially removed by setting all its values to 0. The change in the performance of the model was evaluated using $\Delta R^{2}$, which represents the difference in $R^{2}$ between the original validation dataset and the dataset with the removed columns. This process is repeated for all columns, and the features that result in a decrease in $\Delta R^{2}$ are identified as important, whereas those resulting in an increase in $\Delta R^{2}$ are considered less important. This analysis is performed for each fold, allowing the observation of feature importance across different iterations.

**Figure 3:**
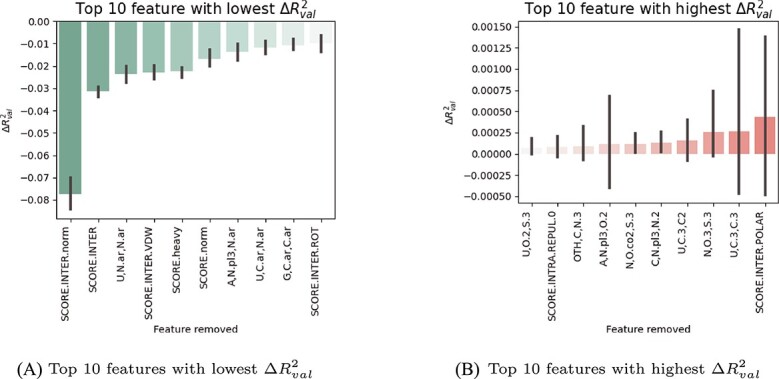
Change in $R^{2}_{val}$ between the original validating dataset and the dataset with the removed column, $\Delta R^{2}_{val}$. Features with $\Delta R^{2}_{val}$< 0 in Figure 2(a) represents a poorer performance of the model after removing the feature, while features with $\Delta R^{2}_{val}$> 0 in Figure 3(b) represents an improvement in performance of the model after removing the feature.

The analysis showed that certain features, such as aromatic interactions represented by the $(U_{N.ar},N.ar)$, $(A_{N.pl3},N.ar)$, $(U_{C.ar},N.ar)$ and $(G_{C.ar},C.ar)$ features, have a significant impact on the model’s predictive capabilities as shown in Figure 3(a). On the other hand, some features yielded positive $\Delta R^{2}$ values as shown in Figure 3(b), indicating that these features negatively affect the accuracy of the model. Despite the recognized importance of polar interactions in NA–ligand systems, the SCORE.INTER.POLAR term, which represents the intermolecular polar interaction score from rDock, showed the lowest importance. This may be due to its redundancy with the more effective capture of similar polar interactions by $F(\frac{1}{r})$ and $F(\frac{1}{r^{6}})$ terms, potentially rendering the SCORE.INTER.POLAR term obsolete. The other low importance features are typically sparse in the dataset. For example, all the features involving highly sparse selenium (Se) atom interactions, with an average of 0.0494% non-0 instances in the dataset, did not reduce the accuracy of the model when they were removed.

The RmsdXNA model incorporates additional interaction terms that are not typically included in other ML models for NA–ligand systems, such as interactions involving metal and modified residue atoms. According to our feature analysis, the average $\Delta R^{2}$ of features containing receptor metals, ligand metals and modified residue atom (atoms with ’OTH’ residues) interactions are $-1.02 \times 10^{-3}$, $-9.92 \times 10^{-4}$ and $-9.87 \times 10^{-4}$ respectively. The negative average $\Delta R^{2}$ values indicate the importance of including these interactions for improved model accuracy. Although these values are minor compared to the $R^{2}$ achieved by the model, keeping these features allows for the incorporation of datasets with rare features in the future, contributing to the model’s ability to learn from diverse information.

### Model performance across data points

The performance of RmsdXNA was assessed by examining its PCC values with respect to different ligand structures. This study aims to understand whether ligands with similar PCC values share common structural features that contribute to their performance variation. The structures of the ligands with the highest and lowest PCC values were selected for analysis.

**Figure 4:**
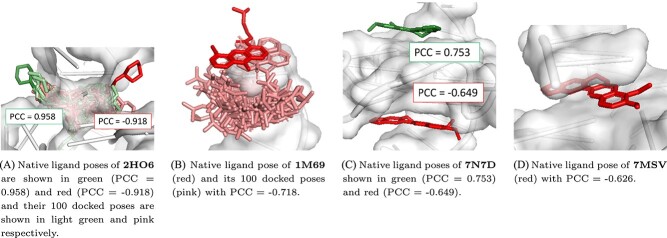
Ligands with high PCC values of the actual vs. predicted RMSD shown in red. For structures that contain multiple ligands, the ligands with high PCC values are shown in green.

Generally, RmsdXNA tends to give lower scores (better rankings) to poses that have more interaction contact with the receptor, such as poses that are deeper in the receptor surface pocket or intercalate between receptor surfaces. Therefore, scoring accuracy decreases for those in which the docking pocket deviates from the native ligand position, such as 2HO6 and 1M69 (Figure 4(a) and 4(b)). In the case of 2HO6, the docked poses from the 2 nearby ligands are generated within the same pocket. However, the results show significant disparity, with one achieving a PCC of 0.958, and the other achieving a PCC of -0.918. This poor performance may be caused by the model using the wrong native ligand pose as a reference when predicting the RMSD of the docked pose. Therefore, removing nearby ligands from the dataset is important for preventing such situations.

In addition, ligands with fused aromatic ring systems that are between the receptor residues tend to have low PCCs, such as 7N7D and 7MSV (Figure 4(c) and Figure 4(d) respectively). From the structure of 7N7D, the ligands that are on the outer surface of the receptor demonstrated better performance than those that are between the receptor surface. This observation suggests that it is difficult for the model to accurately predict the RMSD of docking poses for ligands with extended aromatic rings. One possible explanation is the excessive number of interactions surrounding the ligand, which could introduce noise into the model.

Conversely, ligands with hypoxanthine-like and ruthenium polypyridyl-like complex structures tend to give better results (Table A8). This could be due to the high frequency of such ligands in the dataset, which allows the model to gain better prediction capabilities and understanding of the interactions for this ligand class. Therefore, the performance of the model can be improved by providing a more diverse training dataset.

The ranking capability of RmsdXNA was analyzed for different groups of structures, such as structures solved using NMR vs. X-ray diffraction, and complexes involving DNA, RNA or RNA–DNA hybrid receptors. The average PCC of the ligands for each different group is shown in Table 1. For all the different groups of structures, the PCC and SRCC of RmsdXNA outperformed that of rDock. These findings reinforce the reliability and robustness of RmsdXNA across the different groups.

**Table 1:** The average PCC and SRCC of the actual RMSD vs. RmsdXNA and rDock’s scoring functions of the poses of each ligands are summarized in this table. The PCC and SRCC are categorized by their structure solving method using NMR and X-ray diffraction, and by their receptor types of DNA, RNA and RNA–DNA hybrid, with bold values representing better performance among the scoring functions.

Structure solving	No. of	Average PCC of ligand		Average SRCC of ligand	
method	structures	RmsdXNA	rDock	RmsdXNA	rDock
NMR	178	**0.43016 $\pm $ 0.35800**	0.25019 $\pm $ 0.32280	**0.35788 $\pm $ 0.3453**	0.21064 $\pm $ 0.31150
X-ray diffraction	1256	**0.67580 $\pm $ 0.35295**	0.24267 $\pm $ 0.32234	**0.61679 $\pm $ 0.34101**	0.24267 $\pm $ 0.32234
	**No. of**	**Average PCC of ligand**		**Average SRCC of ligand**	
**Receptor type**	**structures**	**RmsdXNA**	**rDock**	**RmsdXNA**	**rDock**
DNA	575	**0.56084 $\pm $ 0.39992**	0.26301 $\pm $ 0.34507	**0.51012 $\pm $ 0.39238**	0.23920 $\pm $ 0.33165
RNA	841	**0.71087 $\pm $ 0.31031**	0.23422 $\pm $ 0.30581	**0.64222$\pm $ 0.30092**	0.20328 $\pm $ 0.29235
DNA-RNA hybrid	16	**0.38890 $\pm $ 0.51146**	0.05664 $\pm $ 0.26279	**0.37680 $\pm $ 0.50930**	0.074360 $\pm $ 0.25892

### Evaluation on the test dataset

The model is evaluated using a testing dataset consisting of 125 out of 140 PDB structures (Appendix N) from various sources, Yan [46], Ruiz [18], Chen [61] and Philips [62], similar to the test dataset used in Feng et al. [19]. These datasets primarily comprise complexes of DNA and RNA receptors with small molecules and peptide ligands. The remaining 15 PDB structures in the testing dataset were not used because of the selection criteria, as described in Appendix B. In addition, the remaining 1309 PDB structures from the full dataset were used for training the model using the best model’s hyperparameter values.

**Figure 5:**
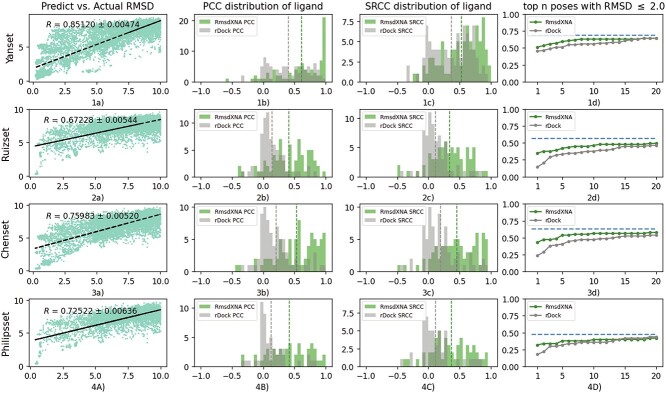
Result of the testing dataset. Figures in column a) shows the predicted vs. actual RMSD of the ligand pose; b) shows the PCC distribution of the ligands; c) shows the SRCC distribution of the ligands; d) shows the fraction of ligands in the top-scored n poses that contains the pose with the lowest RMSD. Row 1, 2, 3 and 4 represents results from the Yan, Ruiz, Chen and Philips dataset respectively.

The results of the evaluation of RmsdXNA’s prediction on the testing datasets are shown in Figure 5. For all the testing datasets, a strong correlation between the predicted RMSD and the actual RMSD of the docked poses of 0.67228 to 0.85120 was obtained. Based on the PCC and SRCC values of each ligand and the fraction of ligands for which the top-n-pose had an RMSD $\leq $ 2.0 Å, RmsdXNA outperformed rDock in terms of ranking capability and pose selection. The ability of each docking tool in selecting close-to-native poses in the top scoring poses was also compared. For PDB structures that were not found in our dataset, it is assumed that RmsdXNA is unable to predict the close-to-native pose for that ligand. For PDB structures containing multiple ligands, the pose that gave the lowest RMSD was used for evaluation. RmsdXNA achieved the highest success rate compared to the other tools for all the testing datasets as shown in Table 2. Overall, RmsdXNA’s scoring accuracy outperforms that of existing docking tools for various unseen datasets and it is more capable of identifying the correct docking pose for NA–ligand complexes.

**Table 2:** Number of PDB structures where the top 1 or top 5 poses selected by the different docking programs in flexible–ligand docking, contain poses with RMSD $\le $ 2 Å on the 4 test sets of diverse NA–ligand complexes. The results from NLDock, AutoDock, rDock (NLDock) and DOCK 6 are obtained from Feng et al. [19]. For RmsdXNA and rDock, only ligands that are selected as described in Section 2.1 are used in this evaluation.AU: Please provide suitable wording for the table footnote to give the meaning of the bold values given in Table 2 directly in the text.

	Top1 pose with rmsd $\le $ 2.0 Å				Top5 pose with rmsd $\le $ 2.0 Å			
Method	Yan	Ruiz	Chen	Philips	Yan	Ruiz	Chen	Philips
RmsdXNA	**37**	**21**	**28**	**14**	**43**	**25**	**32**	**16**
rDock	33	10	15	9	36	21	26	**16**
NLDock	28	9	14	11	36	15	22	15
AutoDock	10	1	3	1	14	2	5	2
rDock (NLDock)	24	8	10	8	29	9	14	10
DOCK 6	24	6	8	5	30	6	9	5

### Scoring power comparison validated by MST experiment

The comparison between rDock and RmsdXNA scores was validated using the in-vitro results of MST on $^{GC}$PAN triplex by Swain et al. [42]. The scores of the best poses selected by each scoring function are summarized in Table A10 and A11. For the hit compounds, RmsdXNA achieved SRCC = 0.39286 and ranks Compound 15 at 4/7, slightly outperforming rDock’s result of SRCC = 0.21429 and ranking Compound 15 at 5/7. Comparing the scores of Compound 15 and its modification counterparts, RmsdXNA ranks Compound 15 at a better rank of 2/4, whereas rDock rank Compound 15 at 4/4. These results validate that RmsdXNA’s ability to distinguish strong from weak binders and its ligand screening capability is better than that of rDock.

**Figure 6:**
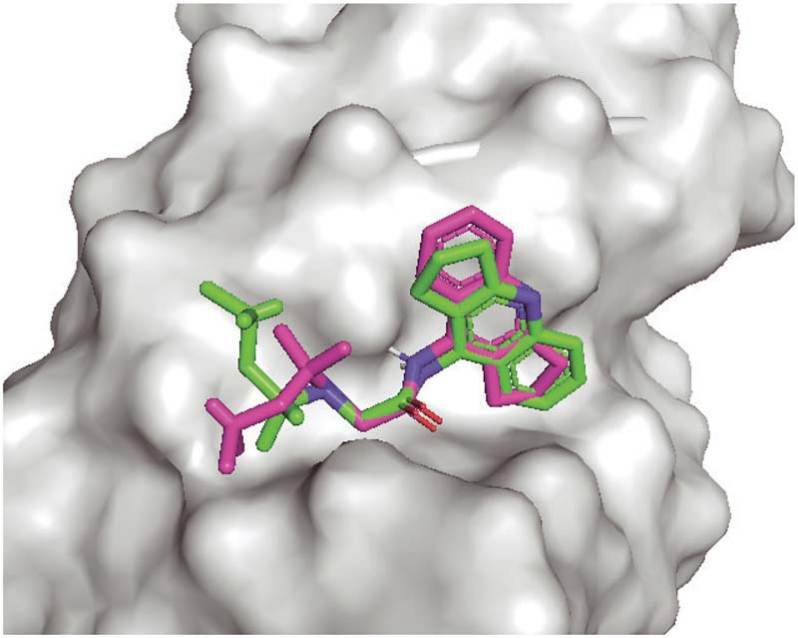
Best pose selected by RmsdXNA (green) and rDock (pink) on $^{GC}$PAN triplex RNA.

The best poses of Compound 15 selected by RmsdXNA and rDock are shown in Figure 6, differentiated by the direction that the extended five carbon ring is pointing: it is pointing away from the receptor for RmsdXNA, but pointing towards the receptor for rDock’s best pose. The MD trajectory of Compound 15 is more stable when using the initial pose selected by RmsdXNA, indicating a closer resemblance between the actual binding mode of Compound 15 and the best pose selected by RmsdXNA. The difference in the stability of the ligand trajectories highlights the importance of selecting initial structures using the docking score to enhance the reliability of MD simulations.

### Screening application validated with MD simulations

**Figure 7:**
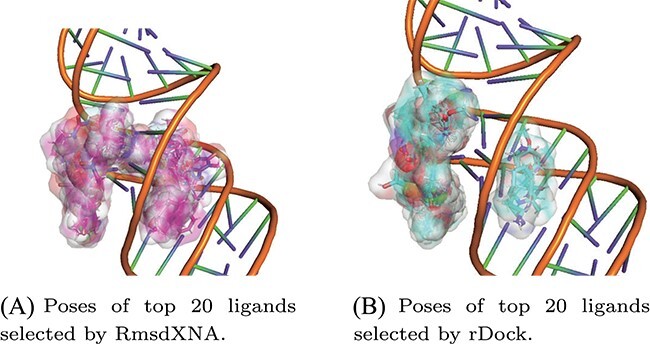
Poses of the top 20 ligand selected by RmsdXNA and rDock from Chembridge and Life Chemicals screening libraries docked on 4PLX using rDock. Top-scored ligand poses selected by RmsdXNA tend to intercalate between the RNA residues. This is not observed for the top-scored ligands selected by rDock.

The screening power of RmsdXNA and rDock for VS application on MALAT1 was compared by evaluating the MD trajectories of the top ligands selected by each scoring function. RmsdXNA was able to identify about 6 times more stable ligands out of 20 top-scored ligands compared to rDock as shown in Table 3. This finding suggests that RmsdXNA has a higher success rate in identifying potential ligands that binds strongly to the receptor, making it more accurate and reliable for screening. Interestingly, no common top candidate ligands were identified by RmsdXNA and rDock. From the top poses selected by RmsdXNA and rDock as illustrated in Figure 7, it is observed that RmsdXNA tends to give better scores to poses that intercalate between the RNA residues, but no such poses are selected by rDock. This indicates that a change in the score function can result in different outputs. The ability to accurately identify stable ligands is crucial for selecting reliable docking tools and refining computational docking protocols. By improving the efficiency and success rate of VS-related efforts in drug discovery, RmsdXNA can greatly impact the field.

**Table 3:** Number of stable ligands from the 20 top-scored ligands selected by RmsdXNA and rDock during the MD simulation of 100 ns. The Chembridge and Life Chemicals screening compound libraries were used for the VS.

Dataset	Number of stable ligands based on MD simulations	
	RmsdXNA	rDock
Chembridge	**13**	2
Life chemicals	**12**	2

### Limitations

In this study, RmsdXNA was evaluated and trained on ligand poses generated by local docking, excluding poses with RMSD $\ge $ 10 Åfrom the native pose. This exclusion may limit the functionality of RmsdXNA when the docking pocket is unknown, but it is necessary to reduce noise in the dataset during training and evaluation [63]. RmsdXNA is expected to perform well if docking tools can accurately generate close-to-native ligand poses or address the flexibility issue of the receptor as discussed in Stefaniak and Bujnicki [36]. Furthermore, tools that identify RNA-ligand binding sites [64], can be used in conjunction with RmsdXNA to accurately identify the docking pocket.

The small number of experimentally determined NA–ligand complexes presents a challenge for improving the ML model for NA docking tools. Infrequent feature interaction also hinder the model from learning this information effectively. However, the advantages of easy feature extraction and adaptability to different datasets presented in RmsdXNA simplify the addition of new data points to enhance the accuracy of the model. While RmsdXNA demonstrated overall better results than rDock in validation against $^{GC}$PAN triplex MST experiment and MD simulations validation of MALAT1 VS, a wider variety of experimental dataset involving diverse receptors is required to comprehensively evaluate the reliability of the scoring functions for virtual screening and pose identification.

## CONCLUSION

RmsdXNA, a ML regression model, was successfully developed and demonstrated to be an effective tool for predicting the RMSD of docked ligand poses in NA complexes. The model incorporates distance-based features that capture atom-atom interactions in NA complexes, including those containing metal ions, modified residues, and peptide ligands. The experiments showed that there is a correlation between the binding affinity and the predicted RMSD of a ligand pose, which can be used for pose identification and screening. RmsdXNA outperformed the other docking tools when evaluated on the testing dataset. It also achieved high prediction accuracy with an average validation $R^{2}$ of 0.68301 $\pm $ 0.02330 and an average PCC of 0.64531 $\pm $ 0.36262 and SRCC of 0.58465 $\pm $ 0.3519 across the different ligands. The alignment of the scoring ranking with the result of the MST experiments conducted on $^{GC}$PAN triplex validated RmsdXNA’s better capability than rDock in distinguishing strong binders from weaker binders. RmsdXNA also has a higher success rate in identifying good ligand candidates for VS, supported by the MD simulations of MALAT1. Overall, this study demonstrated that ML can be utilized to improve the accuracy of molecular docking methodologies in the field of computational drug design.

## Author contributions

L.H. was responsible for carrying out all the experimental work, data collection and analysis. Y.G. provided valuable guidance and advice on the methods used in this project, contributing to the overall design and approach. C.K. contributed expertise in data analysis and machine learning modeling, offering insightful advice throughout the analysis phase. All authors read and approved the final manuscript.

**Key Points**

- The intermolecular atom-atom pairwise distance of docking poses, r, were used to generate $\sum{\frac{1}{r}}$ and $\sum{\frac{1}{r^{6}}}$ features, which were subsequently used to construct the dataset for training the machine learning model. The model, namely RmsdXNA, is trained on 980 nucleic acid (NA)–ligand complex structure dataset using the XGBoostRegressor algorithm to predict the root-mean-square-deviation (RMSD) of a docked pose.
- Modification of the features used in Wang et al. [41] allows us apply these features from protein–ligand complexes to NA–ligand complex systems in RmsdXNA. The results show successful application of RmsdXNA to various NA–ligand complexes, including different types of NA receptors and ligands.
- The RMSD predicted by RmsdXNA outperforms rDock score in pose identification when evaluated on the testing dataset used in Feng et al. [19].
- RmsdXNA demonstrated better scoring power and pose identification than rDock, using the binding affinities of small molecules on polyadenylated nuclear (PAN) triplex RNA obtained by microscale thermophoresis (MST).
- Molecular dynamics (MD) simulation on MALAT1–ligand complex was performed to validate the binding of the top ligand candidates selected by RmsdXNA and rDock during virtual screening. The results showed that RmsdXNA had a higher success rate in selecting stable ligands by approximately 6 times.

## Figures and Tables

**Figure A1:**
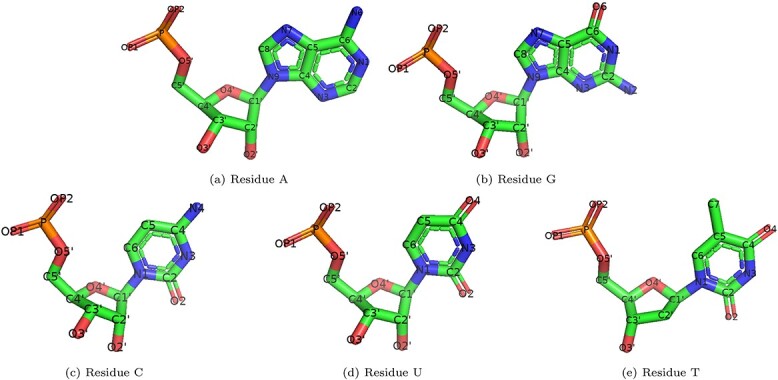
The atom names of residues A, G, C, U/T.

## Data Availability

The data underlying this article are available in the article and in its online supplementary material.

## A Appendix

### Appendix A. PDB list

The list of PDB structures from NDB used for docking are shown in the list below. The PDB IDs in red are not used in the dataset as the ligands were docked unsuccessfully, where there are fewer than 100 poses out of 400 dock attempts with RMSD $\le $ 10Å.

**Table:**

100D	101D	102D	108D	109D	110D	121D	127D	128D	129D	130D	131D	144D	151D	152D	154D
159D	166D	173D	182D	185D	193D	195D	198D	1AGL	1AJU	1AKX	1AL9	1AM0	1AMD	1ARJ	1AW4
1BYJ	1C9Z	1CX3	1D10	1D11	1D14	1D17	1D21	1D22	1D30	1D32	1D36	1D37	1D38	1D43	1D44
1D45	1D46	1D48	1D54	1D58	1D63	1D64	1D67	1D85	1D86	1DA0	1DA9	1DB6	1DCR	1DDY	1DJ6
1DL8	1DNE	1DNH	1DNS	1DPL	1DSC	1DSD	1DVL	1EDR	1EEL	1EHT	1EI2	1EI4	1EM0	1ET4	1EVV
1F1T	1F27	1F6C	1FD5	1FDG	1FMN	1FMQ	1FMS	1FN2	1FQ2	1FTD	1FUF	1FYP	1G3X	1G4Q	1G5L
1GJ2	1I0T	1I1P	1I2X	1I2Y	1I3W	1I5V	1I7J	1I9V	1IMR	1IMS	1J7T	1JO2	1JTL	1JUX	1K2L
1K2Z	1K9G	1KCI	1KGK	1KOC	1KOD	1KVH	1L0R	1L1H	1L1V	1LC4	1LEX	1LEY	1LVJ	1M69	1MP7
1MTG	1MWL	1MXK	1N37	1N7A	1N7B	1NAB	1NBK	1NEM	1NJN	1NTA	1NTB	1NZM	1O15	1O9M	1OE5
1OFX	1OLD	1OVF	1P96	1P9X	1PBR	1PIK	1PQQ	1PRP	1PWF	1Q8N	1Q9X	1QCH	1QD3	1QSX	1QV4
1QV8	1R4E	1R68	1RAW	1S2R	1TN2	1TOB	1UNJ	1UNM	1UTS	1UUD	1UUI	1VRO	1VS2	1VTG	1VTH
1VTI	1VTJ	1VZK	1WOE	1X95	1XCS	1XPF	1XVR	1Y0Q	1Y26	1Y27	1Y84	1Y8L	1YKV	1YLS	1YRJ
1YYO	1Z3F	1Z58	1Z5T	1Z8V	1ZJG	1ZPH	1ZPI	1ZZ5	202D	206D	209D	210D	211D	215D	216D
217D	224D	227D	231D	234D	236D	245D	248D	258D	261D	263D	264D	267D	268D	269D	276D
278D	282D	288D	289D	292D	293D	296D	298D	2A04	2A5R	2ADW	2ARG	2AU4	2B0K	2B2B	2B3E
2B57	2BE0	2BEE	2CKY	2D47	2D55	2DA8	2DBE	2DCG	2DND	2DYW	2EES	2EET	2EEU	2EEV	2EEW
2ELG	2ESI	2ESJ	2ET0	2ET3	2ET4	2ET8	2EZF	2EZG	2F4S	2F4T	2F4U	2F8W	2FCX	2FCY	2FCZ
2FD0	2G5K	2G9C	2GB9	2GCV	2GDI	2GIS	2GJB	2GVR	2GWA	2GYX	2H0W	2H0Z	2HO6	2HO7	2HOJ
2HOM	2HOO	2HOP	2HRI	2I2I	2I5A	2IE1	2JUK	2JWQ	2KD4	2KGP	2KMJ	2KTZ	2KU0	2KX8	2KXM
2L1V	2L7V	2L8H	2L94	2LLJ	2LWH	2LWK	2M4Q	2MB3	2MCC	2MCO	2MG8	2MGN	2MIY	2MS6	2MXS
2N0J	2N6C	2N96	2NEO	2NLM	2NZ4	2O1I	2O3V	2O3W	2O3X	2O3Y	2O43	2O45	2OE5	2OE8	2OEY
2OYQ	2PIK	2PL8	2PWT	2QWY	2R2U	2RF3	2TOB	2TRA	2XNW	2XNZ	2XO1	2YDH	2YGH	2YIE	2Z74
2Z75	302D	303D	304D	305D	306D	308D	311D	316D	319D	320D	321D	323D	324D	326D	328D
336D	352D	355D	358D	360D	367D	375D	385D	386D	3AJK	3AJL	3B4A	3B4B	3B4C	3BNQ	3BNR
3C3Z	3C44	3C5D	3C7R	3CCO	3CDM	3CE5	3D0U	3D2G	3D2V	3D2X	3DIG	3DIL	3DIM	3DIO	3DIQ
3DIR	3DIX	3DIY	3DIZ	3DJ0	3DJ2	3DS7	3DVV	3E5C	3E5E	3E5F	3EGZ	3EM2	3EQW	3ERU	3ES0
3ET8	3EUI	3EUM	3EY0	3EY2	3EY3	3F2Q	3F2T	3F2W	3F2X	3F2Y	3F30	3F4E	3F4G	3F4H	3FO4
3FO6	3FT6	3FU2	3FWO	3FX8	3G4M	3G8T	3G96	3G9C	3GAO	3GCA	3GER	3GES	3GJH	3GJJ	3GOG
3GOT	3GSJ	3GSK	3GX2	3GX3	3GX5	3GX6	3GX7	3I1D	3I5L	3IQN	3IQR	3IRW	3JQ4	3K1V	3L3C
3LA5	3MIJ	3MUM	3MUR	3MUT	3MUV	3MXH	3N4O	3NP6	3NPN	3NPQ	3NX5	3NYP	3NZ7	3OIE	3OMJ
3OZ3	3OZ5	3Q3Z	3Q50	3QCR	3QF8	3QRN	3QSC	3QSF	3RKF	3S4P	3SC8	3SD3	3SKI	3SKL	3SKR
3SKT	3SKW	3SKZ	3SLM	3SLQ	3SUH	3SUX	3T5E	3TD1	3TZR	3U05	3U08	3U0U	3U38	3U4M	3UVF
3UXW	3UYB	3UYH	3V7E	3WRU	403D	407D	410D	428D	432D	442D	443D	447D	448D	449D	452D
453D	454D	459D	465D	473D	474D	482D	4AGZ	4AH0	4AH1	4AOB	4B5R	4BZT	4BZU	4BZV	4D9X
4D9Y	4DA3	4DAQ	4E1U	4E7Y	4E8K	4E8M	4E8N	4E8P	4E8Q	4E8R	4E8S	4E8T	4E8V	4E8X	4E95
4ERJ	4ERL	4F8U	4F8V	4FAQ	4FAR	4FAU	4FAW	4FAX	4FB0	4FE5	4FEJ	4FEL	4FEN	4FEO	4FEP
4FRG	4FRN	4FXM	4G0F	4GPW	4GPX	4GPY	4GQJ	4GXY	4HIF	4HQI	4I9V	4IJ0	4ITD	4JD8	4JF2
4JIY	4K31	4K32	4KQY	4KZD	4L5K	4L81	4LTF	4LTG	4LTH	4LTI	4LTJ	4LTK	4LTL	4LVV	4LVW
4LVX	4LVY	4LVZ	4LW0	4LX5	4LX6	4M3I	4M3V	4MJ9	4MS5	4NFO	4NYA	4NYB	4NYC	4NYD	4NYG
4O5W	4O5X	4OQU	4P1D	4P20	4P3S	4P5J	4P95	4PDQ	4Q9Q	4Q9R	4QIO	4QK8	4QK9	4QKA	4QLM
4QLN	4QVI	4R4A	4R8J	4RE7	4RZD	4TS0	4TS2	4TZX	4TZY	4U8A	4U8B	4U8C	4W90	4W92	4X18
4X1A	4XNR	4XW7	4XWF	4Y1I	4Y1J	4Y1M	4YAZ	4YB0	4YMC	4ZC7	4ZNP	5BJO	5BJP	5BTP	5C45
5C7U	5C7W	5CCW	5CDB	5D5L	5DAM	5DE5	5DHB	5ET2	5FK1	5FK4	5FK5	5FK6	5FKE	5FKF	5FKG
5FKH	5HBW	5HIX	5IP8	5IU5	5IWJ	5JEU	5JEV	5JLW	5KPY	5KRG	5KVJ	5KX9	5L00	5LFS	5LFW
5LFX	5LIG	5LIT	5LWJ	5MVB	5NPM	5O62	5O69	5OB3	5SWE	5T4W	5T83	5TPY	5UED	5UEE	5UX3
5UZA	5V0H	5V0O	5V1L	5V3F	5V9Z	5VCF	5VCI	5VJ9	5VJB	5W77	5XI1	5XJZ	5XZ1	5Z1H	5Z1I
5Z71	5Z80	5Z8F	5ZEI	5ZEJ	6AST	6AZ4	6BFB	6BNA	6BST	6C63	6C64	6C65	6C8E	6C8K	6C8L
6C8M	6C8O	6CB3	6CC1	6CC3	6CCW	6CHR	6CIL	6CK4	6CK5	6D8A	6DB8	6DLQ	6DLR	6DLS	6DLT
6DMC	6DMD	6DME	6DN1	6DN2	6DN3	6DY5	6E1S	6E1U	6E1V	6E1W	6E84	6E8T	6E8U	6FC9	6FZ0
6G7Z	6G8S	6GIM	6GYV	6GZK	6GZR	6HAG	6HBT	6HBX	6HC5	6HMO	6HWG	6IJV	6IJW	6J0H	6J2W
6JBF	6JBG	6JJ0	6JJH	6JJI	6JWD	6JWE	6K3Y	6KFI	6KFJ	6KFL	6KN4	6KXZ	6LNZ	6M4T	6M5B
6M5J	6N5K	6N5N	6N5O	6N5P	6N5Q	6N5S	6N5T	6O2L	6OD9	6P2H	6P45	6PNK	6PQ7	6Q57	6QIV
6QN3	6R6D	6RNL	6RSO	6RTI	6S15	6S7D	6SX3	6T3K	6T3N	6T3R	6T3S	6TB7	6TF0	6TF1	6TF2
6TF3	6TFE	6TFF	6TFG	6TFH	6U6J	6U7Y	6U7Z	6U89	6U8F	6U8U	6UBU	6UC7	6UC8	6UC9	6UP0
6V0L	6V9D	6VA2	6VA3	6VA4	6VMY	6VUI	6VWT	6VWV	6WTL	6WTR	6WZR	6WZS	6XB7	6XCL	6XKN
6XKO	6XRQ	6XT7	6YL5	6YLB	6YMI	6YMJ	6YMK	6YML	6YMM	7A3Y	7ATG	7CPS	7CSK	7D12	7D5E
7D7W	7D7X	7D7Y	7D7Z	7D81	7D82	7DFY	7DJU	7DJV	7DJW	7DWH	7E9E	7E9I	7EAF	7EDL	7EDM
7EDT	7EL7	7ELP	7ELQ	7ELR	7ELS	7EOG	7EOH	7EOI	7EOJ	7EOK	7EOL	7EOM	7EON	7EOO	7EOP
7FHI	7FJ0	7JNH	7KBW	7KBX	7KLP	7KU4	7KUK	7KUL	7KUM	7KUN	7KUO	7KUP	7KVT	7KVU	7KVV
7KWK	7L0Z	7LNE	7LNF	7LNG	7MSV	7N7D	7N7E	7OA3	7OAV	7OAW	7OAX	7OTB	7PNG	7Q7X	7Q7Y
7Q7Z	7Q80	7Q81	7Q82	7RWR	7SZU	7TD7	7TDA	7TDB	7TDC	7TZR	7TZS	7TZT	7TZU	7V9E	7W9N
7WGW	7X2Z	7X3A	7XLV												
1AO1	1LEJ	2RSK	2RU7	4GMA	5DE8	5ODF	5ODM	5OE1	6E8S	6GZ7	6I4N	6LEW	6RIO	7FGT	7RIL

### Appendix B. Data refinement for data preparation

All NA molecules selected using *bymolecule* entity expansion and protein chains with masses greater than 1500 atomic units (au) were labeled as receptors. The protein receptor was removed during docking.

The following data refinements are performed for data preparation:

- The remaining molecules that are not labeled as receptors, excluding solvent molecules$^{\ast }$ or molecules with fewer than 6 heavy atoms, are labeled as ligands.
- A bond is added between an unbonded residue and the NA receptor if the distance between the atoms is $\le $ 1.65 Å to fix the missing bond in the structure, and these residues are treated as the receptors.
- Ligands with a mass larger than 1500 au were removed. This process filters away large ligands or proteins that are highly flexible and may act as noise for the model [63].
- Ligands that were closer than (radius of gyration of ligand + 10 Å) from a protein receptor removed to exclude ligands with conformations that may be affected by nearby protein receptors.
- In cases where the NA-ligand complexes contained multiple ligands, only ligands more than 4.0 Å from each other were used for docking, while the other ligands were removed from the dataset. This is because ligands that are nearby may influence each others’ native pose. The absence of nearby ligands when performing docking may undermine the accuracy of the model in predicting the RMSD of the docked poses. Additionally, identical ligands in close proximity may result in incorrect reference pose being used for predicting the RMSD of the docked pose if the docking pockets are nearby.
- For structures with alternative conformations, the first variant is used for receptor atoms, while complexes with alternative conformations in the ligand are removed. This prevents the problem of having multiple references of the ligand’s native poses when measuring the RMSD of the docked poses.
- Only the first model is used for structures solved using nuclear magnetic resonance.
- Complexes without any valid ligand molecules were excluded from the experiment.

$^{\ast }$ List of residue names of molecules that are labeled as solvent and not used as ligand for docking: 13D, 1PE, ACT, AGU, ARF, BA, BCT, BME, BR, CL, CON, CPT, DMS, EDO, EOH, FMT, GAI, GLY, GOL, GZ6, IPA, IRI, MGX, MOH, NCO, NRU, OHX, PDO, PGE, PO4, PTN, RHD, RUH, SE4, SEY, SO4, TEA, TMO, URE,

### Appendix C. NA and ligand atom groups

**Table A1:** Atom group of the receptor atoms in ’OTH’ residue group based on its SYBYL atom type. The atoms are grouped according to their element, while the halogens are grouped into a ’*Hal*’ group. For modified residues containing ’*O.co2*’ and ’*P.3*’ atoms, since these atoms appear in the backbone and ribose structure (N), ’N’ residue type is assigned to the receptor atoms containing ’*O.co2*’ and ’*P.3*’.

**Atom Group**	**SYBYL atom type**
O	O.2, O.3
C	C.2, C.3
N	N.2, N.3, N.4, N.pl3
Hal	Br, Cl, F, I

Table A1 shows the grouping of the receptor atoms in 'OTH' residue group. For ligand atom type, *C.cat* (carbocation in a guadinium group) and *C.2* (carbon sp2) SYBYL atom types are grouped together to form *C2* atom group. *C.cat* in the ligand only appeared in 6 PDB structures, therefore binning the features formed by C.cat ligand can reduce the sparsity of the dataset and improve the result.

**Table A2:** Atom types considered in feature generation. The SYBYL atom types of the canonical residues is based on the atom name of the atom as shown in Appendix E. *Italicized* atom types are grouped by the methods above shown in Appendix C. 24 ligand atom types for ligand and 26 NA atom types (unique combinations of the atom types that appear in each residue type) were used, resulting in 624 pair of interactions.

**Receptor**		**Ligand**
Residue	Atom type	Atom type
A	N.ar, C.ar, N.pl3	*C2*, C.1, C.3, C.ar, Br, Cl, F, I, N.1, N.2, N.3, N.4, N.am, N.ar, N.pl3 O.2, O.3, O.co2, S.2, S.3, S.O2, P.3, Se, Metal
G	N.ar, C.ar, N.pl3, O.2	
C	N.ar, C.ar, N.pl3, O.2	
U/T	N.ar, C.ar, O.2, C.3	
N	P.3, C.3, O.3, O.co2	
OTH	*O*, *C*, *N*, *Hal*, Se, S.3	
Metal	Metal	

After grouping the receptor and ligand atom types as mentioned above, the atom type representations are shown in Table A2. Columns with all 0 values were removed from the dataset and used to train the model.

### Appendix D. Residue type generalization

The generalization of modified NA residues into the unmodified NA residues based on structural similarity. For each modified residue structure, the atoms that are similar to the ”parent” nucleotide in terms of its position are represented in sticks (thicker) and the atoms are represented by SYBYL atom type for feature generation. On the other hand, the other modified atoms are represented in lines (thinner) and the atoms are represented by their element for feature generation. Visualization of the structures was performed using PyMol.

For new residues that are not shown in this section, similar residue generalization and atom representation methods can be applied for feature generation.

### Appendix E. Atom name and SYBYL atom type of residues A, C, G, U/T

$RES$ of receptor atom that are in the canonical nucleobase position is the classified RNA’s nucleobases group; $RES$ of receptor atom in the ribose and phosphate backbone is ’N’; $RES$ of the metal atoms in the receptor is ’Metal’, while $RES$ for the other atoms is ’OTH’. $rec$ is represented by the SYBYL atom type based on its position as shown in Figure A1 and Table A3.

**Table A3:** The SYBYL atom type representation of the atoms in nucleobases A, C, G, U/T and the backbone (N) based on the atom’s position as shown in Figure A1

Residue	SYBYL atom type	Atom name
A	N.ar	N1, N3, N7, N9
	C.ar	C2, C4, C5, C6, C8
	N.pl3	N6
G	N.ar	N1, N3, N7, N9
	C.ar	C2, C4, C5, C6, C8
	O.2	O6
	N.pl3	N2
C	N.ar	N1, N3
	C.ar	C2, C4, C5, C6
	N.pl3	N4
	O.2	O2
U/T	N.ar	N1, N3
	C.ar	C2, C4, C5, C6
	O.2	O2, O4
	C.3	C7
N	P.3	P
	C.3	C5’, C1’, C2’, C3’, C4’
	O.3	O5’, O3’, O2’, O4’
	O.co2	OP1, OP2

### Appendix F. Hyperparameter values for the best performing model

max_depth: 13, n_estimators: 900, min_child_weight: 5, learning_rate: 0.02, subsample: 0.3, colsample_bytree: 0.4, reg_alpha: 1, reg_lambda: 1, gamma:1, rate_drop: 0.3,

### Appendix G. Best result of other ML models

**Table A4:** The best $R^{2}_{val}$ obtained for different algorithms. All algorithms perform worse than XGBoost, which has a $R^{2}_{val}$ = 0.68301.

Algorithm	Best $R^{2}_{val}$
Multilayer Perceptron	0.43258
AdaBoost	0.41016
Random Forest	0.65553
Support Vector Regression	0.57900

### Appendix H. MD simulation protocol

To prepare for the MD simulations, the force fields of the receptor and ligands were generated using AMBER [65]. Then, the AMBER topologies are converted to GROMACS [66] format using ANTECHAMBER [67] and ParmEd software [68] (https://github.com/ParmEd/ParmEd). MD simulations of the MALAT1-ligand complex in water with 0.15 M sodium chloride ions at 300 K and the TIP3P water model [69] were performed using GROMACS simulation software to generate a 100 ns trajectory.

### Appendix J. Ablation studies results - cutoff distance

**Table A5:** Average validating $R^{2}$ of actual vs. predicted ligand pose RMSD values, $R^{2}_{val}$, trained on datasets generated using cutoff distance of 4 to 10 Å for feature generation. The $R^{2}_{val}$ value and its standard deviation (std) is calculated from the validating $R^{2}$ obtained from each fold in the 5-fold cross-validation training. nfeatures shows the number of features in the dataset, and the % 0 in dataset measures the sparsity of the dataset.

**cutoff distance, $R_{c}$**	**nfeature**	**% 0 in dataset (%)**	** $R^{2}_{val}$ **	**std**
4	1040	92.1	0.64660	0.02483
5	1062	89.2	0.66621	0.02018
6	1072	86.9	0.67458	0.01980
7	1082	84.7	0.67742	0.01882
8	1088	83.1	**0.68301**	0.02330
*9*	1092	82.0	0.67929	0.01941
10	1098	81.3	0.68176	0.02061

### Appendix K. Ablation studies results - feature combinations

**Table A6:** Average validating $R^{2}$ of actual vs. predicted ligand pose RMSD values, $R^{2}_{val}$, trained using datasets generated using combinations of rDock, $F(\frac{1}{r})$, $F(\frac{1}{r^{6}})$ and $F(\frac{1}{r^{12}})$ features. rDock feature represent the components generated from rDock scoring function, while the $F(\frac{1}{r})$, $F(\frac{1}{r^{6}})$ and $F(\frac{1}{r^{12}})$ features represents the features used to describe the intermolecular force field energy as shown in Section 2.3.

Feature	nfeature	$R^{2}_{val}$	std
rDock	30	0.48328	0.03950
rDock + $F(\frac{1}{r})$	559	0.66988	0.02106
rDock + $F(\frac{1}{r^{6}})$	559	0.6737	0.02028
rDock + $F(\frac{1}{r^{12}})$	559	0.66803	0.0210
*rDock + $F(\frac{1}{r})$ + $F(\frac{1}{r^{6}})$ (default)*	1088	**0.68301**	0.02330
rDock + $F(\frac{1}{r})$ + $F(\frac{1}{r^{12}})$	1088	0.67798	0.01998
rDock + $F(\frac{1}{r^{6}})$ + $F(\frac{1}{r^{12}})$	1088	0.67601	0.01964
rDock + $F(\frac{1}{r})$ + $F(\frac{1}{r^{6}})$ + $F(\frac{1}{r^{12}})$	1619	0.67910	0.01763
$F(\frac{1}{r})$ + $F(\frac{1}{r^{6}})$	1060	0.65344	0.02865
$F(\frac{1}{r})$ + $F(\frac{1}{r^{6}})$ + $F(\frac{1}{r^{12}})$	1591	0.65241	0.02956

### Appendix L. Ablation studies results - feature binning

**Table A7:** Average validating $R^{2}$ of actual vs. predicted ligand pose RMSD values, $R^{2}_{val}$, for the best models from datasets generated by different atom representations. The receptor and ligand atoms are represented in either default (Appendix C), element, or SYBYL MOL2 format. The highest average validating $R^{2}$ of 0.68301 is obtained for the default dataset.

**Modification name**	**nfeature**	** $R^{2}_{val}$ **	**std**	**Atom representation**	
				**Receptor**	**Ligand**
*atomgroup (default)*	1088	**0.68301**	0.02330	Default	Default
ligandelem	516	0.67128	0.02350	Default	Element
ligandmol2	1126	0.68173	0.0206	Default	SYBYL MOL2
recelem	850	0.67447	0.02168	Element	Default
recmol2	1174	0.68209	0.0184	SYBYL MOL2	Default
ligandrecelem	408	0.66726	0.02244	Element	Element
ligandrecmol2	1256	0.67945	0.01654	SYBYL MOL2	SYBYL MOL2

### Appendix M. Ligand analysis

**Table A8:** List of ligands with similar structures and relatively high PCC between the actual RMSD of the poses and RmsdXNA predicted RMSD.

(a) List of PDB IDs with structure containing ruthenium polypyridyl-like complex ligands and its PCC values.		(b) List of PDB IDs with structure containing hypoxanthine-like ligands and its PCC values	
**PDB ID**	**PCC of ligand**	**PDB ID**	**PCC of ligand**
4R8J	0.99890	4FEN	0.99755
4LTF	0.99748	4FEL	0.99568
5LFW	0.99559	7V9E	0.99272
4LTJ	0.99521	7Q7Y	0.99260
3UYB	0.99455	2EEU	0.99238
4E8S	0.99202	4FEJ	0.99174
4E1U	0.98948	2EET	0.98871
5LFX	0.98790	1Y27	0.98822
4M3V	0.98708	7Q80	0.98757
4JD8	0.98595	4FE5	0.98755

### Appendix N. Testing data list from NLDock

The list of PDB IDs from Feng et al. [19] that are used for the testing data are shown in the list below. The PDB IDs in red are not used in the testing dataset and are missing from the dataset, due to the filter criteria when selecting the PDB structures. Table A9 shows the number of NA-ligand structures in the test dataset and the number of ligands that are used for the evaluation of RmsdXNA.

**Table:**

1AJU	1AKX	1AM0	1ARJ	1BYJ	1DB6	1EHT	1EI2	1F1T	1F27	1FMN
1FYP	1I9V	1J7T	1KOC	1KOD	1LC4	1LVJ	1MWL	1NBK	1NEM	1NTA
1NTB	1NZM	1O15	1O9M	1P96	1PBR	1Q8N	1QD3	1QV4	1QV8	1R4E
1TOB	1UTS	1UUD	1UUI	1XPF	1Y26	1Y27	1YKV	1YRJ	1ZZ5	2AU4
2B57	2BE0	2BEE	2D55	2ESI	2ESJ	2ET3	2ET4	2ET8	2F4S	2F4T
2F4U	2FCX	2FCY	2FCZ	2FD0	2G9C	2GDI	2GIS	2JUK	2JWQ	2KGP
2KTZ	2KU0	2KX8	2L94	2MB3	2O3V	2O3W	2O3X	2OE5	2OE8	2PWT
2TOB	2XNW	2XNZ	2XO1	2YDH	2YGH	2Z74	2Z75	316D	3C44	3D2X
3DIL	3DS7	3DVV	3E5C	3F2Q	3FO4	3FO6	3FU2	3G4M	3GAO	3GER
3GES	3GOG	3GOT	3GX2	3GX3	3GX5	3GX7	3LA5	3MUM	3MUR	3MXH
3NPN	3NPQ	3Q3Z	3Q50	3S4P	3SD3	3SLM	3SUX	407D	4AOB	4ERJ
4FE5	4JF2	4KQY	4LVV							
1CVX	1CVY	1F4S	1FJG	1G5K	1HNW	1NYI	1U8D	1XBP	2G5Q	2LOA
2OGN	2XO0	3OWZ	408D							

**Table A9:** The full number of PDB structures in the NA-ligand test dataset and the number of ligands that were used in our dataset.

**Dataset**	**No. of structures in dataset**	**No. of structures used**
**Yan**	77	69
**Ruiz**	56	53
**Chen**	56	54
**Philips**	42	37

### Appendix O. Ranking of compounds based on their binding affinity and scores

**Table A10:** Binding affinities of hit compounds by Swain et al. [42] and the RmsdXNA and rDock scores of the best pose selected by each scoring method. The compounds are also ranked based on their binding affinity and scores.

**Compound**	**Binding Affinity**	**Binding Affinity rank**	**RmsdXNA score**	**RmsdXNA rank**	**rDock score**	**rDock rank**
8	700 $\mu $M	5	5.974	2	-18.572	3
13	305 $\mu $M	4	5.387	1	-18.678	2
15	27 $\mu $M	1	6.415	4	-18.153	5
18	3.2 mM	7	6.865	6	-18.336	4
19	800 $\mu $M	6	6.988	7	-13.614	7
20	128 $\mu $M	2	6.319	3	-19.418	1
25	209 $\mu $M	3	6.836	5	-16.083	6

**Table A11:** The RmsdXNA and rDock scores and score ranking of the best pose selected by each scoring method for Compound 15 and its modification counterparts.

**Compound**	**RmsdXNA score**	**RmsdXNA rank**	**rDock score**	**rDock rank**
15	6.415	2	-18.153	4
15-A1	6.621	3	-18.841	3
15-A2	6.616	4	-25.707	1
15-A3	6.247	1	-21.201	2
